# A numerical investigation of electric field effects on spray jet flames with charged fuel droplets

**DOI:** 10.1177/17568277261431227

**Published:** 2026-04-22

**Authors:** Navraj S Lalli, Andrea Giusti

**Affiliations:** 1 Department of Mechanical Engineering, 4615 Imperial College London, London, UK

**Keywords:** Large-eddy simulations, charge injection atomisation, electric field control, fuel flexibility, spray penetration, flame characteristics

## Abstract

The use of electrostatic fields to control the location of charged fuel droplets in a spray jet flame is investigated as a means of developing fuel-flexible combustion systems to accelerate the transition to carbon-neutral transportation. The focus of this work is on the effects of an external electric field parallel to the jet direction on the spray penetration and fuel vapour distribution, and the subsequent effects on flame shape and location. This study is conducted using large-eddy simulations under the assumption that there is negligible production of charged species during combustion. Non-reacting simulations at atmospheric conditions show that with practical electrostatic fields and droplet charges, it is possible to significantly change the spray penetration. Electric forces in the opposite direction to the spray jet lead to a reduction in the spray penetration, an effect that increases with stronger electrostatic fields. This leads to an increase in drag between the droplets and the air flow, resulting in a smaller penetration of the air jet. The reduced droplet penetration confines the fuel vapour to a region closer to the jet inlet and leads to a more compact flame in the corresponding reacting cases. Similar trends are observed in simulations performed at a higher pressure and temperature. This study suggests that electrostatic fields can be used with charged fuel droplets to provide an element of control over spray jet flames, which may allow for the development of novel hybrid thermal–electric combustion systems.

## Introduction

An urgent transition to a zero-carbon energy infrastructure is needed to reduce the impact of human activities on the concentration of carbon dioxide in the atmosphere and on climate change. An analysis of carbon dioxide emissions by sector highlights that the transportation sector is responsible for a significant portion of global carbon dioxide emissions, approximately 23% in 2023 with aviation constituting over 10% of these emissions.^1^ Additionally, estimates from pre-pandemic data indicate that aviation accounts for around 5% of human-induced global warming.^2^ Decarbonising the transportation sector is therefore essential and requires the use of alternative energy vectors, including batteries and zero-carbon fuels, as well as the development of new technologies for their safe and efficient use. Although progress towards a zero-carbon economy has been made for both power generation, for example, through the development of technologies for zero-carbon fuels,^3^ and land transportation, with electrification being the main solution to abate carbon emissions,^4^ challenges still remain for the aviation sector. In particular, the specific energy density of the latest battery technology is insufficient to allow for all-electric long-haul flights.^5^ Furthermore, the necessity of keeping the weight and size of the aircraft down, together with safety considerations, makes the use of zero-carbon fuels such as hydrogen challenging. Therefore, it is not surprising that the main engine and aircraft manufacturers are currently looking at hybrid thermal–electric propulsion configurations^6^ coupled with sustainable aviation fuels (SAFs) as a medium-term solution to lower the carbon footprint of aviation. The use of hybrid thermal–electric propulsion systems will lead to a large availability of electrical energy on board, which could offer new possibilities to achieve control over the combustion process and to extend the range of fuels used in aviation.^7^ In this context, technologies that make use of electrical energy to improve mixing^8,9^ and combustion characteristics^10^ have been proposed.

In general, the behaviour of a combustion system used in aviation is very sensitive to the type of fuel. In most cases, an injection system and a combustor designed to work for a given fuel lose performance (e.g. lower atomisation quality and higher emissions at the exhaust) when operated with fuels with significantly different properties. Consequently, SAFs are typically designed with thermophysical properties that replicate the properties of conventional jet fuel so that they can be used in existing combustion technologies.^7,11^ However, this may exclude some fuels that have good properties from an environmental standpoint because the combustor design is not suitable for their use. From this perspective, it would be ideal to have a fuel-flexible combustor, that is, a combustor that can operate well with fuels characterised by a wide range of properties. By taking advantage of the electrical energy available on board in hybrid configurations, Fredrich et al.^8^ proposed the combination of charge injection atomisation and electrostatic control of droplet trajectories to achieve fuel pre-evaporation in a compact space. Considering the same technology, Giusti and Fredrich^12^ further investigated the possibility of pre-evaporating fuels characterised by different properties, such as n -decane, n -heptane and ethanol. Their study concluded that it is possible to control the trajectories of droplets of different fuels and achieve their pre-evaporation with electric fields of similar strengths. Although the focus of those studies was exclusively on pre-evaporation technologies, such work is an important step towards the development of new combustion technologies that utilise electric fields to provide fuel flexibility.

The possibility of using electric fields to control combustion is not limited to the modulation of mixing of charged fuel droplets. Electric fields can also be used to directly affect flame characteristics. Over the years, many studies, mainly experimental, have demonstrated that both AC and DC electric fields can influence emissions, change the shape of flames and allow for the control of combustion instabilities.^13–17^ The macroscopic effect of electric fields observed in those investigations is mainly due to the action of the electric field on charged species that form during the combustion process, leading to an ionic wind.^18^ In the context of spray flames with no applied electric field, recent studies have shown that the presence of charged droplets can affect the local flame structure.^19^ However, the combination of an external electrostatic field and charged droplets in a reacting environment has not been investigated yet. In this case, the applied electrostatic field is expected to affect both the positions of the fuel droplets, which influences the mixing of fuel vapour with the surrounding gaseous mixture, and the transport of ionic species. The combination of these effects will change the flame characteristics.

The aim of this work is to investigate the use of electrostatic fields to manipulate the location of fuel in a spray jet flame containing charged fuel droplets, a configuration of practical interest for both power generation and transportation, and to evaluate the subsequent effects on mixing and the reactive field. The specific objectives of this work are (i) to investigate the effect of an electric field parallel to the jet axis on the location of charged droplets under non-reacting conditions and (ii) to study the spray penetration, vapour distribution and flame location under reacting conditions for different strengths of the external electric field and different pressure conditions. The investigation is performed using large-eddy simulations under the assumption that there is negligible formation of charged species during the combustion process. This assumption means that electric field effects on the transport of ionic species and electrons generated by the combustion process are not accounted for, which allows for a first assessment of electric field effects on the mixing and reacting field due solely to the modulation of droplet locations. The combined effect of an electric field on the spray and on ionic species and electrons formed in the reacting region should be investigated in future work.

## Methods

### Configuration

**Figure 1.**
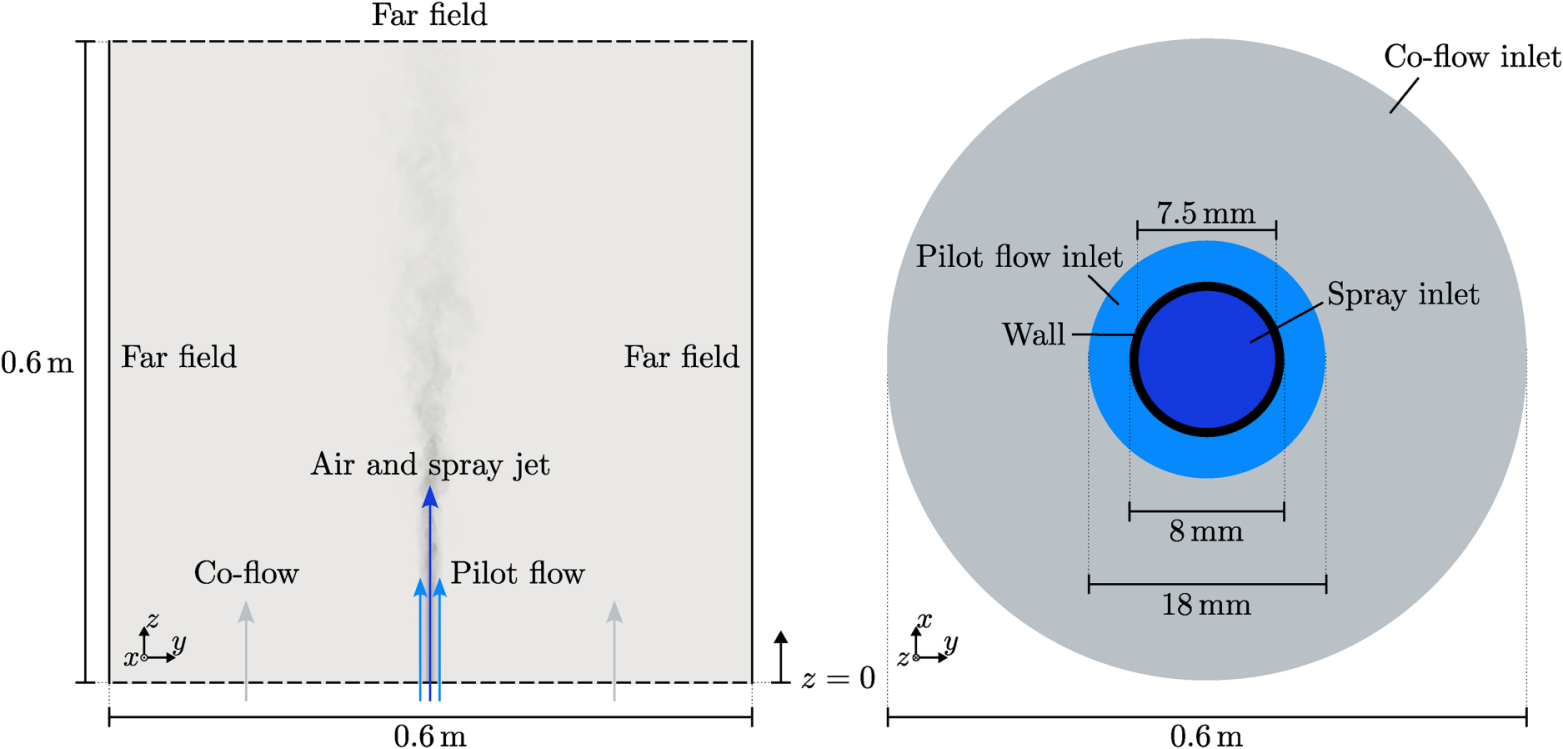
A schematic of the configuration investigated in the present work, which indicates the dimensions of the domain used for large-eddy simulations. A vertical cross-section of the domain is shown on the left; the inlet flows are shown on the right.

The spray jet flame schematically shown in Figure 1 is investigated. The configuration is similar to the piloted spray flame investigated at the University of Sydney.^20^ The spray, generated upstream of the burner, is injected together with an air flow through a central duct of diameter 7.5 mm, referred to as the spray inlet. This duct is surrounded by a concentric flow, called the pilot flow, that under reacting conditions enables the injection of hot combustion products to stabilise the flame. The pilot flow is surrounded by a co-flow of air. In the configuration investigated in this study, the co-flow extends over the remainder of the bottom boundary of the domain. In experimental set-ups, however, the co-flow is often injected through a smaller annular duct.

The electric field is generated by applying a potential difference between two planar electrodes with normal parallel to the axis of the spray duct (the z -axis). One of these electrodes is located at the height of the spray inlet (i.e. z=0 – see Figure 1 for the definition of the coordinate system), whereas the other electrode is located further downstream, sufficiently far from the flame. These electrodes can be practically implemented by means of two metallic grids or perforated plates (e.g. Park et al.^18^). The surfaces of the electrodes are assumed to be sufficiently large to generate a uniform electric field in the entire flame zone, with the electric field direction parallel to the axis of the spray duct. The investigated fuel is kerosene, which is a dielectric liquid with a static relative electric permittivity of approximately 2 at room temperature.^21^ Charged droplets of dielectric liquid fuels can be generated using charge injection atomisation.^22,23^ It is assumed here that the charged spray of kerosene fuel droplets is produced by charge injection atomisation in a configuration that results in negatively charged fuel droplets.

### Investigated cases

The effects of an electrostatic field on the spray and flow dynamics are investigated under both non-reacting and reacting conditions. The non-reacting (‘cold-flow’) cases allow the effects of an applied electric field on the trajectory of droplets and spray jet dynamics to be studied without the additional complexity of chemical reactions. The reacting cases build on the knowledge developed from the cold-flow conditions and reveal how modifying droplet trajectories using an electric field affects the fuel vapour distribution and flame characteristics. Cold-flow simulations are performed at a pressure of 1 bar, with air injected through all inlets at a temperature of 293 K. For the reacting cases, two pressures are investigated, p=1 bar and p=5 bar. The case of higher pressure is intended to replicate the conditions in the combustion chamber of a gas–turbine cycle operated at ground level. Therefore, the temperature of the air inlets is set to 483 K, a value obtained by assuming a compression of air from ambient conditions with isentropic efficiency equal to 0.9. The higher pressure case allows for an investigation of the electrostatic field effects on the spray jet flame under conditions much closer to practical applications. The investigated cases are summarised in Table 1. Note that for the cold-flow simulations the pilot flow is pure air at the same temperature as the other air flows, whereas for the reacting cases the pilot flow consists of a mixture of combustion products (mass composition: $Y_{{\text{CO}}_{2}}=0.151$ , $Y_{{\text{H}}_{2}\text{O}}=0.124$ , $Y_{{\text{N}}_{2}}=0.725$ ), taken from complete stoichiometric combustion of methane with air under the conditions used in the large-eddy simulations. The behaviour of the system is assessed for different strengths of the electrostatic field; electrostatic fields pointing both upwards (positive z -direction) and downwards are investigated. Since the droplets are negatively charged, an upward electric field results in a downward electric force on the droplets. A monodisperse spray is assumed with droplet diameter at injection equal to 50 µm and a mass flow rate corresponding to a stoichiometric air–fuel ratio in the spray jet ( 0.030g/s at p=1 bar and 0.089g/s at p=5 bar). Droplets are injected with a temperature of 300K and a velocity of 10m/s in the z -direction. The charge of the droplets is assumed to be half of the Rayleigh stability limit,^12^ which experiments suggest is an upper limit for the droplet charge after atomisation.^24^

**Table 1.** Investigated conditions.

Case	p (bar)	$U_{j}$ (m/s)	$U_{\mathrm{cf}}$ (m/s)	$U_{p}$ (m/s)	${\dot{m}}_{f}$ (g/s)	$T_{\mathrm{air}}$ (K)	$T_{p}$ (K)	$E_{z}$ (kV/m)
Non-reacting	1	8.2	0.5	3.7	0.030	293	293	{ − 100, 0, 50, 100}
Reacting A	1	8.2	0.5	3.7	0.030	293	2326	{ − 100, 0, 50, 100}
Reacting B	5	8.2	0.5	3.7	0.089	483	2446	{ − 100, 0, 50, 100}

p is the pressure; $U_{j}$ is the bulk velocity of the jet; $U_{\mathrm{cf}}$ is the velocity of the co-flow; $U_{p}$ is the velocity of the pilot flow; ${\dot{m}}_{f}$ indicates the mass flow rate of the spray; $T_{\mathrm{air}}$ and $T_{p}$ are the temperatures of the air flow and pilot flow, respectively; and $E_{z}$ is the z -component of the applied electric field (all other components are zero).

### Large-eddy simulations

Simulations are performed with the large-eddy simulation approach and a two-way coupled Eulerian–Lagrangian method for dilute sprays. The solver, implemented in OpenFOAM-v7,^25^ is the same as used in previous work^8^ with the addition of chemical source terms for the gaseous phase and combustion modelling. The charged fuel droplets are modelled as point Lagrangian particles with gravity, drag and electric forces acting on them; all other forces are neglected. A drag force relation for spherical droplets is adopted.^9^ Local variations of the electrostatic potential due to the presence of charged droplets and Coulomb repulsion between the charged droplets are neglected. Therefore, the electric force acting on the droplets is simply computed as the product of the droplet charge and the external electric field. Secondary atomisation, including droplet breakup induced by electrostatic repulsion between the charges inside each droplet,^21,26^ is not accounted for in the present work. Secondary breakup models that account for droplet deformation and the charge distribution within each droplet under an external electric field should be developed in the future.^21^ The presence of charges within the droplets is assumed not to affect the evaporation process.^27^ In addition, it is assumed that the charge of each droplet stays constant during the entire evaporation process.^28^ Under cold-flow conditions, the evaporation time of the droplets (approximately 5.1 s, computed with single-droplet simulations using the methodology discussed in Fredrich and Giusti^29^) is much larger than the simulated time. Therefore, non-evaporating droplets are used in the cold-flow simulations. In the reacting simulations, evaporation is modelled using a rapid mixing model with the Ranz–Marshall heat transfer correlation.^30^ Furthermore, combustion is modelled with infinitely fast chemistry and a single-step chemical mechanism, with water and carbon dioxide being the only combustion products. This simple modelling approach is sufficient to provide an estimate of the flame location, which is the focus of this work. The mechanism used does not include the formation of charged species, such as ionic species, charged radicals and free electrons; therefore, it is not possible to capture the influence of the applied electric field on the transport of gaseous species or related ionic wind effects.^31,32^ An extensive investigation of the flame structure using a more comprehensive combustion model and a detailed chemical mechanism that includes the formation of charged species should be performed in future work. This will allow for a complete assessment of electric field effects on the flame structure and pollutant formation, as well as an examination of electric field effects on flame behaviour under extreme conditions, such as flame extinction and re-ignition. Depending on the chosen combustion model and simulation framework, closure of terms in the transport equations representing sub-grid effects of the electric field may be necessary.

### Numerical set-up

The numerical domain, shown in Figure 1, is a cylindrical geometry concentric with the axis of the spray duct, with the lower boundary located at the exit of the spray jet. The velocity of the air flow entering through the spray inlet is modelled with a power-law profile ( $u_{z}=U_{0}(1-r/R{)}^{1/7}$ , where $U_{0}$ is the velocity at the centre of the air flow, r is the radial distance from the jet axis and R is the radius of the spray jet inlet). In contrast, a uniform velocity is used for both the pilot flow and the air co-flow. No turbulent fluctuations are imposed at any of the inlets. A wave transmissive condition is used for the pressure at the far field boundaries. The composition of each species and the temperature are imposed at all inlets. Droplets are injected at the spray inlet boundary, with the injection uniformly distributed over the boundary. For the electrostatic potential, a potential difference is imposed between the top and bottom boundaries of the domain. The bottom boundary of the domain is grounded (set to zero potential), while the value of the potential at the top boundary is adjusted to obtain the desired external electric field: the external electric field in the z -direction is given by $E_{z}=-\phi_{\mathrm{top}}/L$ , where $\phi_{\mathrm{top}}$ is the electric potential imposed at the top boundary of the domain and L is the distance between the top and bottom boundaries ( L=0.6 m). The domain is discretised with a hexahedral grid of approximately 4.9 million cells. Refinements are used near the inlets and in the shear layers between the three concentric flows. Second-order spatial discretisation schemes are used for all quantities and a first-order Euler scheme is used for the discretisation in time. The timestep (of the order of $10^{-6}$ s) was adjusted to keep the maximum Courant-Friedrichs-Lewy number below 0.4. For the computation of statistics, time-averages in the non-reacting and reacting cases were performed for 0.1 and 0.5 s, respectively. All post-processing was performed using Python with PyVista.^33^

## Results

The velocity field and spray location obtained with cold-flow simulations are first presented to assess electric field effects in the absence of a flame. Then, reacting conditions are analysed with a focus on the fuel vapour distribution and the flame location.

### Cold-flow simulations

**Figure 2.**
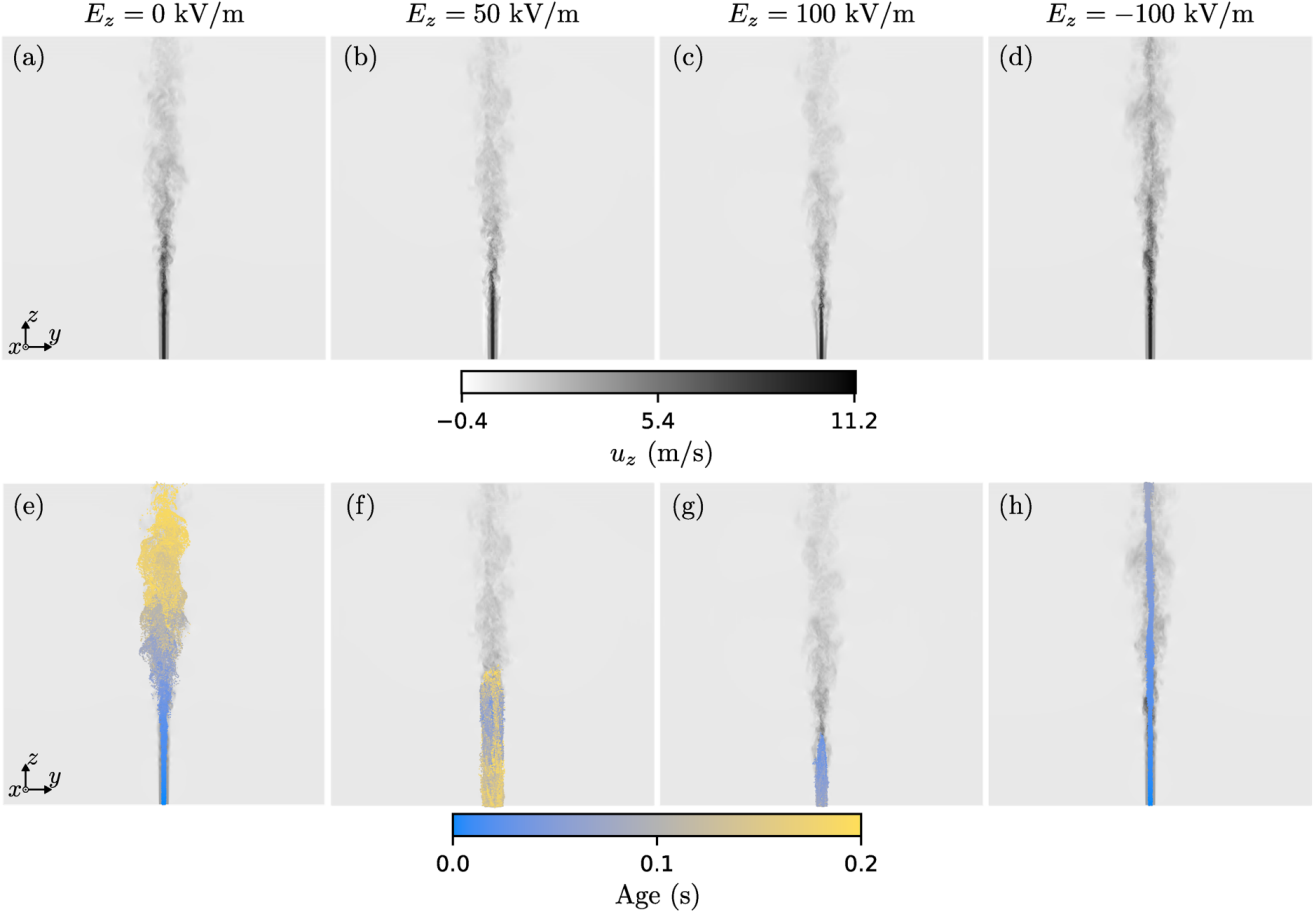
The z -component of the jet velocity (a–d) and the cloud of charged droplets (e–h), coloured by age, overlaid on the z -component of the jet velocity after time t=0.2 s from the beginning of droplet injection. The velocity fields are shown on a plane normal to the x -direction passing through the centre of the domain. Each column corresponds to a different electric field, as indicated by the annotation at the top of the figure.

Figure 2 shows a snapshot of the spray and the z -component of the jet velocity after 0.2 s from the start of droplet injection for the four electric fields investigated in this work. The age of the droplets is also indicated to show their residence time in the domain after injection. The air flow injected through the spray inlet exhibits the typical features observed for jets with a relatively low injection velocity. In the absence of an external electric field, the air jet penetrates up to about one-third of the domain before it breaks up with substantial formation of turbulent structures. The droplets closely follow the evolution of the air flow, that is, with a small relative velocity. This is because the droplets have a relatively small diameter, leading to short dynamic relaxation times. As shown in Figure 2(e), the droplets move vertically upwards in the region immediately downstream of the injection plane and become more dispersed as the jet develops into turbulent structures. When an electric field is applied in the positive z -direction (electric forces on the droplets in the negative z -direction), the penetration of the droplets is reduced. The spray penetrates up to a maximum height without reaching the upper boundary of the domain. With an electric field of $E_{z}=50$ kV/m, some of the droplets located in the central region, where the flow velocity is higher, stabilise at an intermediate height of around z=0.25 m. This is clear from the large age of droplets located at the tip of the spray cloud in Figure 2(f). For a given droplet diameter and charge, the electric field strength necessary to stabilise the droplets, that is, zero absolute velocity in the z -direction, is a function of the gas-phase velocity.^12^ Therefore, stabilisation can only be achieved for those droplets in regions of the flow where the air velocity allows for a balance between drag and electric forces. If the air velocity is too small, the electric forcing becomes dominant and a reverse flow of the charged droplets is observed. This is the case for droplets in the outer part of the spray that move back towards the inlet plane (e.g. see the droplets with large age close to the inlet for $E_{z}=50$ kV/m in Figure 2(f)). In principle, the stabilised droplets have an infinite residence time in the domain. However, if evaporation was included in these simulations, the droplet diameter would decrease over time and the subsequent decrease in drag would eventually cause a departure of these droplets from their stabilised positions. If the strength of the electric field is further increased to $E_{z}=100$ kV/m, the motion of the droplets is completely reversed and the droplets quickly return to the inlet plane after an initial transient due to the vertically upward injection velocity. In practical situations, such as a cold start, this situation should be avoided since it leads to entrainment of liquid fuel into the air flows or wetting of the base plates with fuel. Consequently, the electric field strength may need to be modulated during ignition transients. With $E_{z}=-100$ kV/m, the electric forces are in the same direction as the air jet and the spray quickly penetrates and leaves the domain, as shown in Figure 2(h). In this case, the spray tends to stay coherent with little dispersion of the cloud for the entire height of the domain. This is due to the strong vertical acceleration of the droplets induced by the electric forcing, which is dominant over the drag.

**Figure 3.**
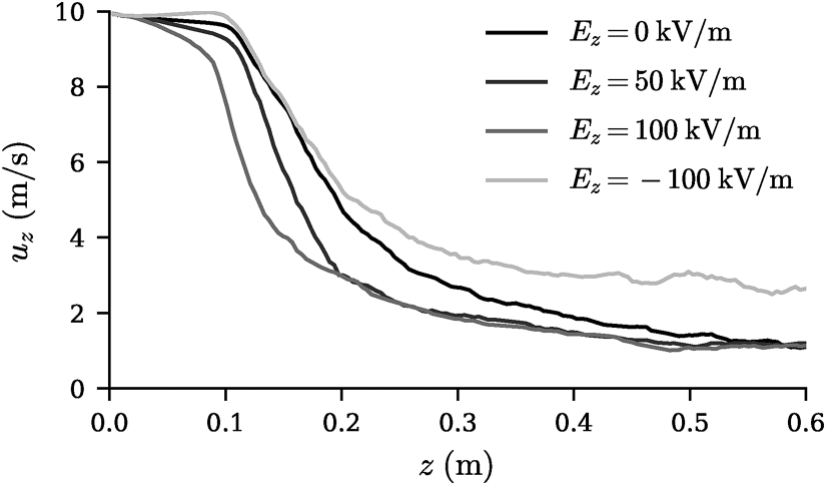
The time-averaged z -component of the gas-phase velocity along a line in the z -direction passing through the centre of the domain. The time average was performed for 0.1 s, starting at 0.2 s from the beginning of droplet injection. Each line corresponds to each of the four cases shown in Figure 2.

The effect of the droplets on the air flow was also investigated. Considering the cases with an applied electric field in the positive z -direction, an increase in electric field strength leads to an increase in momentum loss of the air. This is because stronger positive electric fields result in larger relative velocities between the droplets and the air flow, with some droplets moving in the opposite direction. The interaction between the droplets and the air flow results in turbulent structures forming earlier and a reduced penetration of the air jet, as shown in the top row of Figure 2. This is also demonstrated by Figure 3, which shows that the time average of the z -component of the gas-phase velocity along the centreline of the domain falls with increasing $E_{z}$ . In contrast, when electric forces are in the same direction as the air jet, there is a transfer of momentum from the droplets to the air flow. This increases the speed of the air jet compared to the zero-field case, as is clear from Figure 3. The air jet then tends to stay more coherent and penetrates more compared to the zero-field case (see the top row of Figure 2).

### Flame simulations

**Figure 4.**
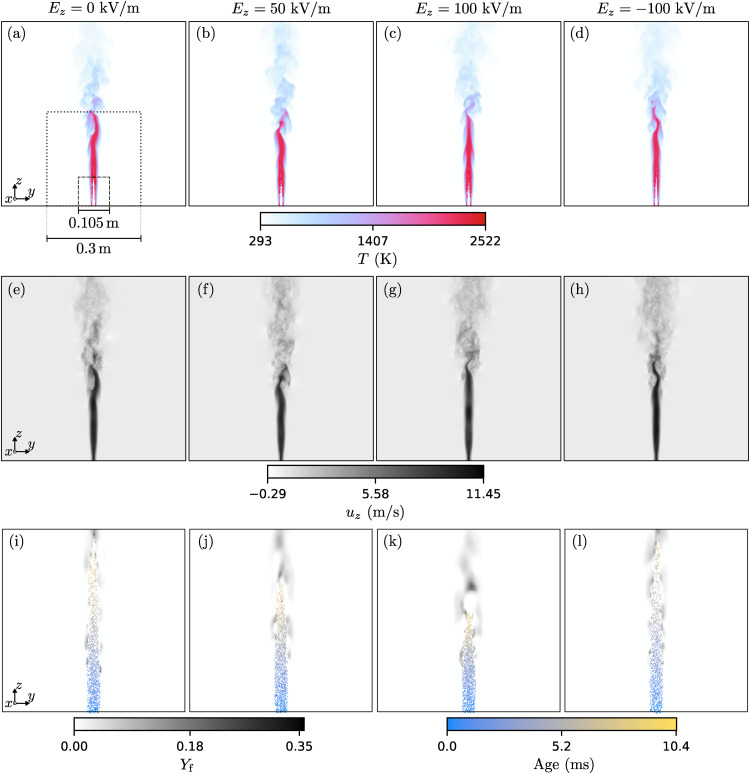
The temperature (a–d), z -component of the velocity (e–h) and an enlarged view of the charged droplets (i–l), coloured by age, overlaid on the mass fraction of fuel, $Y_{f}$ , at time t=0.7 s after the beginning of injection for p=1 bar. The region shown in (i–l) aligns with the dashed square of length 0.105 m marked on (a). The dotted square of length 0.3 m indicates the region visualised in Figure 5. All values are shown on a plane normal to the x -direction passing through the centre of the domain. Each column corresponds to a different electric field, as indicated by the annotation at the top of the figure.

Snapshots of the temperature and z -component of the velocity of the gas phase, together with the location of the spray and the age of the droplets, are shown in Figure 4 for p=1 bar and the different electric fields investigated in the present work. All cases show the formation of a relatively compact flame. Due to the absence of turbulent fluctuations at the inlet, the flow tends to be laminar close to the spray jet inlet, with turbulent structures developing at the tip of the high-temperature region. This behaviour is typical of spray flames under the investigated conditions and is not commented on further. The focus is instead on the spray behaviour and the flame characteristics with different electric fields. As illustrated in Figure 4, all droplets fully evaporate in a region very close to the injection plane (before z=0.105 m) within 10.4 ms from injection, which is a consequence of the high evaporation rates induced by the high flame temperature. For the spray penetration, observations similar to those made for the cold-flow simulations also apply to the reacting cases. For electric fields in the positive z -direction (electric forces on the droplets in the negative z -direction), the penetration of the spray decreases with increasing strength of the electric field. Due to the very short evaporation time, reverse droplet motion is only observed for a very small fraction of droplets (0.05% and 0.3% of the droplets in the domain for $E_{z}=50$ kV/m and $E_{z}=100$ kV/m, respectively). When the applied electric field is in the negative z -direction, the droplets tend to reach the flame region quicker (with a smaller age compared to the case with no applied electric field). However, this does not necessarily lead to a larger spray penetration since droplets fully evaporate before they can reach larger heights. For example, it is evident from Figure 4(i) and 4(l) that the spray penetration is similar for $E_{z}=-100$ kV/m and no external electric field.

**Figure 5.**
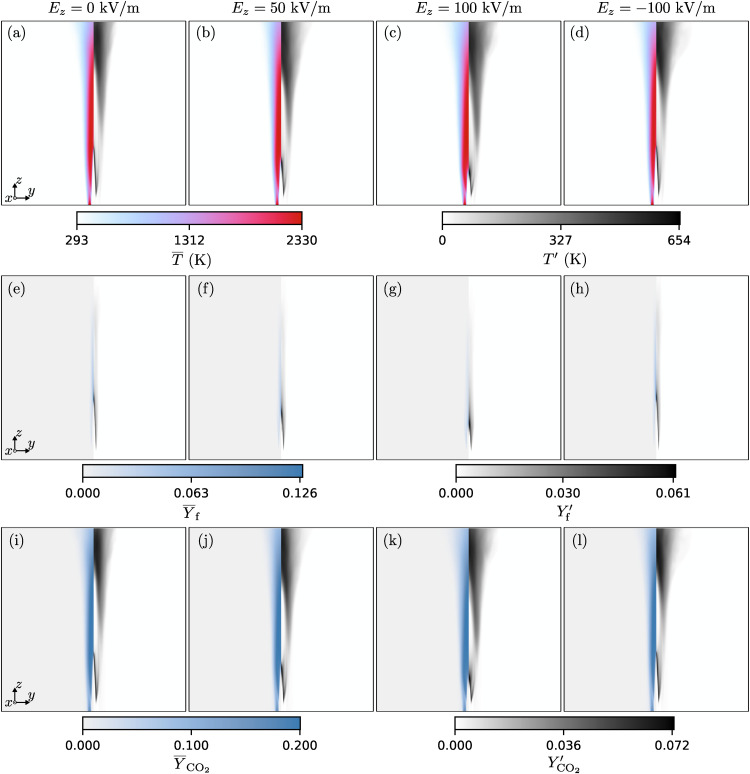
The time-averaged statistics of the temperature (a–d), the mass fraction of fuel (e–h) and the mass fraction of ${\text{CO}}_{2}$ (i–l) at p=1 bar. Each panel shows the time average on the left, which is denoted by T¯ , and the root mean square (RMS) deviation on the right, which is denoted by ${}^{\prime }$ . All values are shown on a plane normal to the x -direction passing through the centre of the domain. The area shown in each panel corresponds to the square of length 0.3 m indicated in Figure 4(a) (dotted line). Each column corresponds to a different electric field, as indicated by the annotation at the top of the figure.

The change in penetration of the spray affects the location of fuel vapour release, altering the extent of the reacting region. This is shown in Figure 5 for p=1 bar by means of the time-averaged fields of temperature, fuel and ${\text{CO}}_{2}$ mass fractions and their root mean square (RMS) deviations about the time-averaged values. When the applied electric field is in the positive z -direction, the reacting layer (the area close to the inlet with high temperature gradients and high RMS values of the temperature) moves closer to the injection plane with increasing strength of the electric field. This is a consequence of the more compact spray penetration and the release of fuel vapour closer to the spray inlet, as shown by the statistics for the fuel mass fraction. With the electric field in the reverse direction, $E_{z}=-100$ kV/m, the extent of the reacting region is similar to the zero-field case because the spray penetration is similar in these two cases. For all cases, the mass fraction of carbon dioxide follows the same trend observed for temperature, as is expected with infinitely fast chemistry and a single-step mechanism. Relatively large fluctuations of the temperature and the carbon dioxide mass fraction are observed in a narrow region of conical shape close to the spray injection plane, which corresponds to the reacting layer, as well as in the post-flame region, where turbulent structures develop.

**Figure 6.**
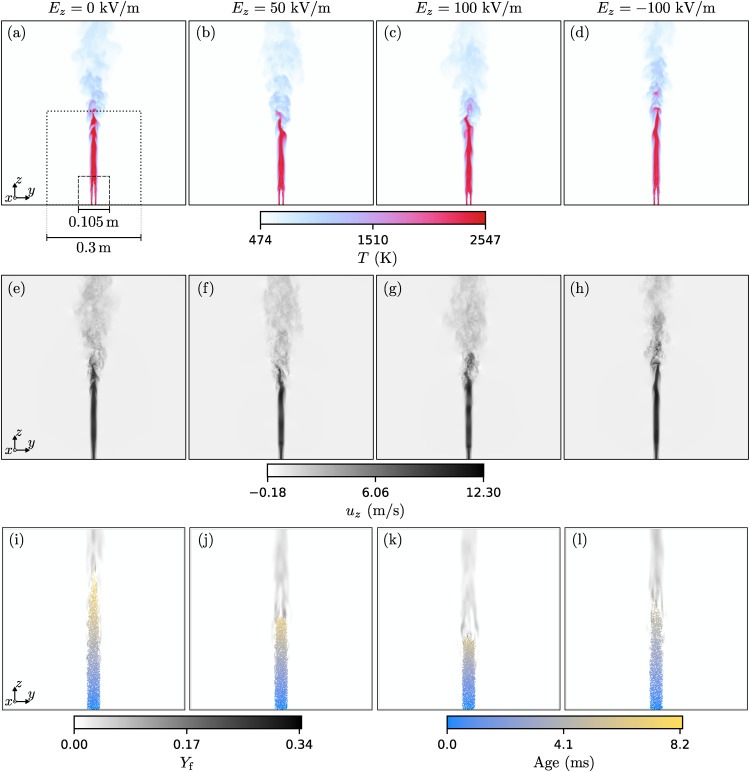
The temperature (a–d), z -component of the velocity (e–h) and an enlarged view of the charged droplets (i–l), coloured by age, overlaid on the mass fraction of fuel, $Y_{f}$ , at time t=0.7 s after the beginning of injection for p=5 bar. The region shown in (i–l) aligns with the dashed square of length 0.105 m marked on (a). The dotted square of length 0.3 m indicates the region visualised in Figure 7. All values are shown on a plane normal to the x -direction passing through the centre of the domain. Each column corresponds to a different electric field, as indicated by the annotation at the top of the figure.

**Figure 7.**
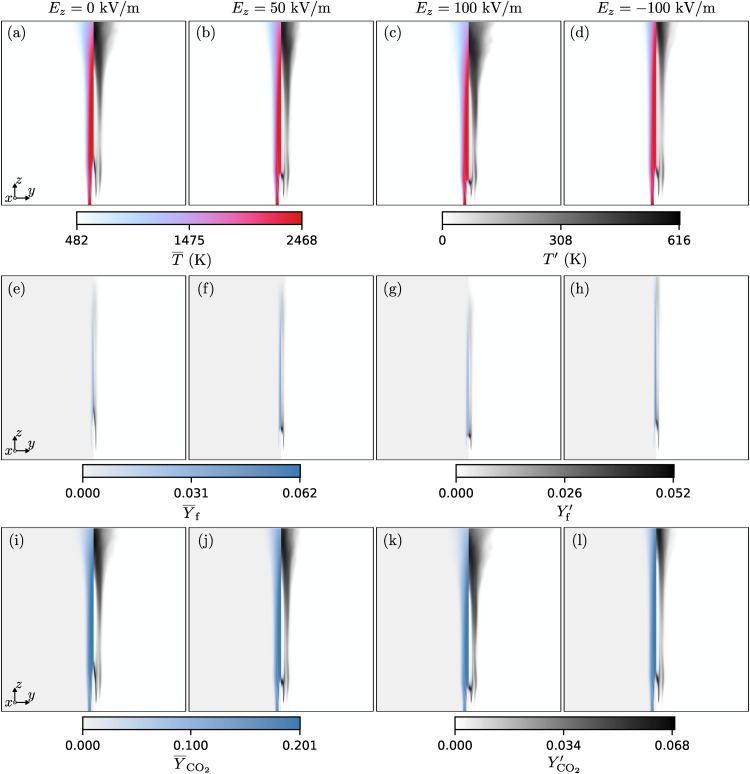
The time-averaged statistics of the temperature (a–d), the mass fraction of fuel (e–h) and the mass fraction of ${\text{CO}}_{2}$ (i–l) at p=5 bar. Each panel shows the time average on the left, which is denoted by T¯ , and the root mean square (RMS) deviation on the right, which is denoted by ${}^{\prime }$ . All values are shown on a plane normal to the x -direction passing through the centre of the domain. The area shown in each panel corresponds to the square of length 0.3 m indicated in Figure 6(a) (dotted line). Each column corresponds to a different electric field, as indicated by the annotation at the top of the figure.

Figures 6 and 7 present the instantaneous and time-averaged results for the cases at p=5 bar. As indicated by the spray location and droplet age reported in Figure 6, evaporation occurs significantly faster at p=5 bar compared to p=1 bar due to the higher temperature of both the air and pilot flows (see Table 1). For example, with no external electric field, droplets completely evaporate within ≈8.2 ms after injection at p=5 bar and within ≈10.4 ms after injection at p=1 bar. The faster evaporation reduces the penetration of the spray for all four electric field configurations investigated in this work, resulting in a more compact flame compared to the lower pressure case. This is further supported by the time-averaged fields and their RMS values provided in Figure 7. As far as the effect of the electric field is concerned, in alignment with the observations at p=1 bar, the height of the reacting region decreases as the electric field $E_{z}$ is increased from 0 to 100 kV/m. With $E_{z}=-100$ kV/m, the reacting layer is located at a height intermediate between the heights of the reacting layers for $E_{z}=0$ kV/m and $E_{z}=50$ kV/m. This suggests that an electric force in the same direction as the spray jet flow does not necessarily lead to a downstream movement of the reacting region. This is due to the strong coupling between the temperature field and the spray evaporation: an upward electric force drives the droplets into the high-temperature flame region, causing a reduction in their lifetime and therefore a lower spray penetration.

**Figure 8.**
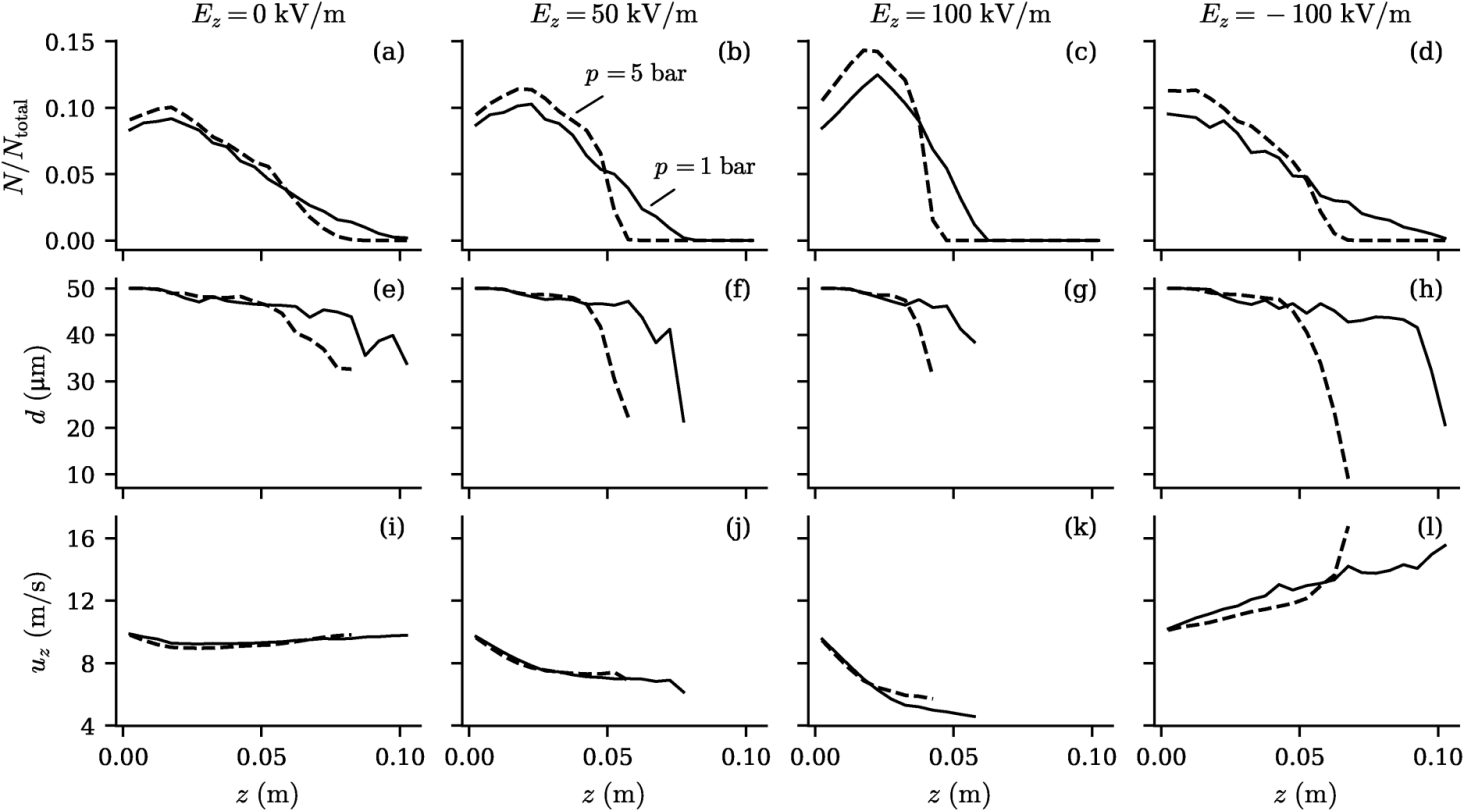
The droplet counts normalised by the total number of droplets in the system (a–d), the droplet diameters (e–h) and the z -component of droplet velocity (i–l) vs. z -position at time t=0.7 s after the beginning of injection. The solid and dashed lines are used to indicate results at p=1 bar and p=5 bar, respectively. Each quantity was computed by arithmetically averaging over droplets in cylindrical bins of height 5 mm in the z -direction.

The droplet dynamics are further analysed in Figure 8, which shows the normalised droplet count, the average diameter and the average axial droplet velocity as a function of the distance from the injection location for the two pressures and the different electric fields investigated in this work. The normalised droplet counts (see Figure 8(a) to (d)) provide further evidence of the reduced penetration of the spray with increasing operating pressure. It is also evident that the spray penetration falls with increasing $E_{z}$ from 0 to 100 kV/m for both pressure conditions. The average droplet diameters (see Figure 8(e) to (h)) tend to be smaller at the tip of the spray in all cases, which is consistent with minimal reverse spray flow. Compared to the zero-field case, the z -component of the droplet velocities (see Figure 8(i) to (l)) is reduced when the electric forcing is in the opposite direction to the spray jet flow and is increased when the electric forcing is in the same direction as the spray jet flow. The decreasing droplet diameters contribute to the change in droplet velocities observed with increasing distance from the injection location since a reduction in droplet diameter increases droplet acceleration in the direction of the electric force (the electric force remains constant with evaporation since constant charge is assumed, while the drag force tends to decrease with decreasing droplet diameter). The high velocity of the droplets at the tip of the spray with $E_{z}=-100$ kV/m supports the conclusion that these droplets tend to penetrate faster into the flame region, possibly resulting in a reduction of the height of the reacting region compared to the zero-field case. This phenomenon should be further investigated in future work with a comprehensive methodology that includes a more accurate description of the flame structure.

## Discussion

The present investigation demonstrates that with realistic electric fields, that is, electric fields with strength significantly below the dielectric strength of air ( ≈3 MV/m at ambient temperature), it is possible to change the spray location and thus influence the fuel vapour distribution and the flame characteristics. The penetration of the spray can be controlled by modulating the strength and direction of the electric field, which may increase the operating range of novel hybrid thermal–electric combustion technologies.

A monodisperse spray with the same charge for all droplets has been considered in this work. The presence of a polydisperse spray with a relatively wide droplet size distribution, together with differences in the charge of each droplet, may make it more challenging to control the penetration of the spray and where the fuel vapour is released. This is because the diameters and charges of the droplets impact the drag and electric forces acting on the droplets, which determine the penetration of the spray. In addition, charge-induced secondary breakup may lead to a significant redistribution of charge from a given droplet to smaller child droplets,^34^ causing further dispersion of the droplet size and charge distributions. Therefore, the control of sprays characterised by a dispersion of diameters and charges should be investigated in future work, while also accounting for charge-induced secondary breakup.^21^ This first requires a more comprehensive characterisation of the sprays produced by charge injection atomisers. Furthermore, this study did not account for repulsive Coulombic interactions between the charged fuel droplets, which may affect the flame shape by increasing the dispersion of the spray. Further work is necessary to accurately model and characterise the effects of electrostatic interactions between the droplets.

The ability to modulate the spray location is strongly dependent on the spray jet bulk velocity. To counterbalance drag forces with electric forces while keeping the required electric field strength below the dielectric strength of air, relatively low jet velocities must be employed. In addition, evaporation occurs significantly faster when increasing the operating pressure from 1 to 5 bar due to the larger air and pilot flow temperatures. Since faster evaporation limits the time available to modify the trajectories of droplets, stronger electric fields will likely be needed to induce significant changes in the spray penetration as the evaporation time is reduced. An alternative would be to inject droplets with a larger diameter to increase their evaporation time. This strategy should be investigated in future research. Additionally, the uniform electric field investigated in this study would be difficult to achieve inside a realistic combustor. Consequently, future studies should move towards the investigation of configurations of practical interest, which may also involve pressures greater than 5 bar when considering medium- to large-sized gas turbine systems.

It should be re-iterated that the chemical mechanism used in the present study does not include the formation of charged species and free electrons. However, there is strong evidence that the combustion of hydrocarbons leads to substantial formation of ionic species.^17,32^ The movement of charged gaseous species under an electric field and their collisions with neutral species could generate a relatively strong flow, known as ionic wind, which could further affect the flame shape. Integration of a detailed chemical mechanism that includes free electrons and ionic species,^31,32^ as well as the development of a large-eddy simulation framework with closure of sub-grid scale terms representing the effects of species drift driven by an electric field, are part of current research. In addition, combustion was modelled in this study with a simple single-step mechanism and with infinitely fast chemistry. The study of pollutant formation and the effects of electric fields on the local flame structure necessarily requires the use of more detailed chemical mechanisms. Furthermore, the investigation of spray flames with a higher level of turbulence will require the use of more advanced combustion models.^35^ Finally, experiments in canonical configurations such as the one studied in this work should be conducted in the future to provide experimental evidence of electric field effects on spray flames with charged droplets and to support the validation of modelling frameworks.

## Concluding remarks

The use of electrostatic forcing to modulate the penetration of charged fuel droplets in a spray jet flame configuration and the subsequent effects on the fuel vapour distribution and the flame shape have been investigated using large-eddy simulations. The results demonstrate that realistic electric fields with strengths of the order of 100 kV/m can provide an element of control over droplet trajectories. When the applied electric field exerts a force on the charged droplets in the opposite direction to the spray jet flow, the penetration of the spray decreases with increasing strength of the electric field. This results in a more compact reacting region that moves closer to the spray inlet. Electric forces in the same direction as the spray jet flow tend to move the droplets away from the spray inlet, an effect that is very evident under cold-flow conditions. This, however, does not necessarily result in flame elongation. An increase in the operating pressure reduces spray penetration and makes the flame more compact, with the applied electric field leading to similar effects on the spray penetration and position of the reacting region as compared to atmospheric conditions. The present investigation provides evidence that electrostatic fields have the potential of assisting fuel preparation by reducing the space necessary for full evaporation, an effect that could be used to build compact combustors with fuel flexibility enabled by modulating the strength of applied electric fields.
